# Growth hormone treatment improves the development of follicles and oocytes in prepubertal lambs

**DOI:** 10.1186/s13048-023-01209-y

**Published:** 2023-07-05

**Authors:** Kexiong Liu, Luyao Zhang, Qi Qi, Junjin Li, Fengxiang Yan, Jian Hou

**Affiliations:** State Key Laboratory of Animal Biotech Breeding, College of Biological Sciences, China Agricultural University, Yuan-Ming-Yuan West Road, Haidian District, Beijing, 100193 China

**Keywords:** Growth hormone, Prepubertal lamb, Oocytes, Ovary, Granulosa cells, Follicular fluid

## Abstract

**Background:**

When prepubertal lambs are superovulated, the ovarian response to gonadotropin stimulation has great individual difference and the collected oocytes have lower developmental ability than that of adult ewes. Over the years, growth hormone (GH) has been used in assisted reproduction because it can improve the reproductive performance in humans and animals. However, the effect of GH on ovaries and oocytes of prepubertal lambs remains unclear.

**Methods:**

Before and during follicle-stimulating hormone (FSH) superovulation of prepubertal lambs (4‒6-week-old), the lambs were treated with high (50 mg) or low dose (25 mg) of ovine GH in a long (5 days) or short (2 days) period. The recovered oocytes were used for in vitro maturation and fertilization, and several parameters of oocyte quality and development capacity were evaluated. The possible underlying mechanisms of GH action were explored by analysis of granulosa cell (GC) transcriptome, ovarian proteome and follicular fluid metabolome.

**Results:**

Treatment of lambs with 50 mg GH over 5 days (long treatment) potentially promoted the response of lambs to superovulation and improved the development capacity of retrieved oocytes, consequently increasing the high quality embryo yield from lambs. A number of differently expressed genes or proteins were found in ovaries between GH-treated and untreated lambs. Cellular experiments revealed that GH reduced the oxidative stress of GCs and promoted the GC proliferation probably through activation of the PI3K/Akt signaling pathway. Finally, analysis of follicular fluid metabolome indicated that GH treatment altered the abundance of many metabolites in follicular fluid, such as antioxidants and fatty acids.

**Conclusions:**

GH treatment has a beneficial role on function of lamb ovaries, which supports the development of follicles and oocytes and improves the efficiency of embryo production from prepubertal lambs.

**Supplementary Information:**

The online version contains supplementary material available at 10.1186/s13048-023-01209-y.

## Background

In vitro production (IVP) of embryos from oocytes of juvenile animals, known as juvenile in vitro embryo transfer (JIVET), is a promising technology to be applied in rapid breeding of livestock species [1]. An advantage of using juvenile lambs (4‒8-week-old) as oocyte donors is that a great number of oocytes can be recovered from ovaries following gonadotropin superstimulation of the animals [1–3]. However, lamb oocytes usually exhibit lower developmental ability than oocytes from adult ewes when they are used for in vitro embryo production [4–8]. To improve the developmental competence of lamb oocytes, many studies have focused on optimizing the in vitro maturation medium by adding various agents to the medium [9, 10]. Nevertheless, the oocyte competence is largely dependent on the development during growth within ovarian follicles [11]. Therefore, treatment of lambs before oocyte retrieval may enhance the development potential of oocytes.

Growth hormone (GH) plays an important role in maintaining normal fertility by exerting biological effects on folliculogenesis, oocyte maturation and steroid synthesis through autocrine or paracrine secretion [12, 13]. Therefore, GH has already been applied as an adjuvant to enhance human ovarian sensitivity to gonadotropin stimulation since 1980s [14]. For instance, treatment with GH can significantly shorten the gonadotropin stimulation duration before oocyte retrieval and increase the number of competent oocytes and clinical pregnancy rate in poor ovarian responders [15, 16]. In sheep, the utility of GH has been reported in estrus synchronization and superovulation of adult ewes for enhancing the follicle development and embryo production [17–22]. These studies support the potential role of GH in improvement of the reproductive performance in mammals.

Although the efficacy of GH on reproduction has been confirmed in adult females in human or animals, it remains to be determined whether GH can improve the ovarian response and oocyte quality of prepubertal animals in the JIVET program. To address this issue, in this study we attempted to treat lambs with GH in the process of superovulation and evaluated its effects on the development of follicles and oocytes in lambs. We found that the ovarian response and oocyte developmental ability could be improved when the lambs were treated with GH. We also explored the possible underlying mechanisms of GH action by analysis of GC transcriptome, ovarian proteome and follicular fluid (FF) metabolome.

## Methods

All chemicals were purchased from Sigma-Aldrich (St. Louis, MO, USA) unless specifically stated.

### Animals and hormonal treatment

A total of 54 female Merino lambs (4‒6-week-old, still on their dams) were selected for the experiments. All lambs together with their mothers were house-fed under conventional management conditions and maintained in Aohan Sheep Breeding Farm, Chifeng, Inner Mongolia, China (42^°^18′ N, 119^°^50′ E). The animal experiments were conducted from July to June (the temperature ranged from 10℃ to 25℃). The protocol of superovulation treatment of lambs was as described previously [9, 10]. Briefly, each lamb received 6 × 45 IU injections of follicle-stimulating hormone (FSH) (Ningbo Sansheng Pharmaceutical, China) given at approximately 12 h intervals, and 400 IU of equine chorionic gonadotropin (eCG; Ningbo Sansheng Pharmaceutical) given at the first FSH administration.

**Fig. 1:**
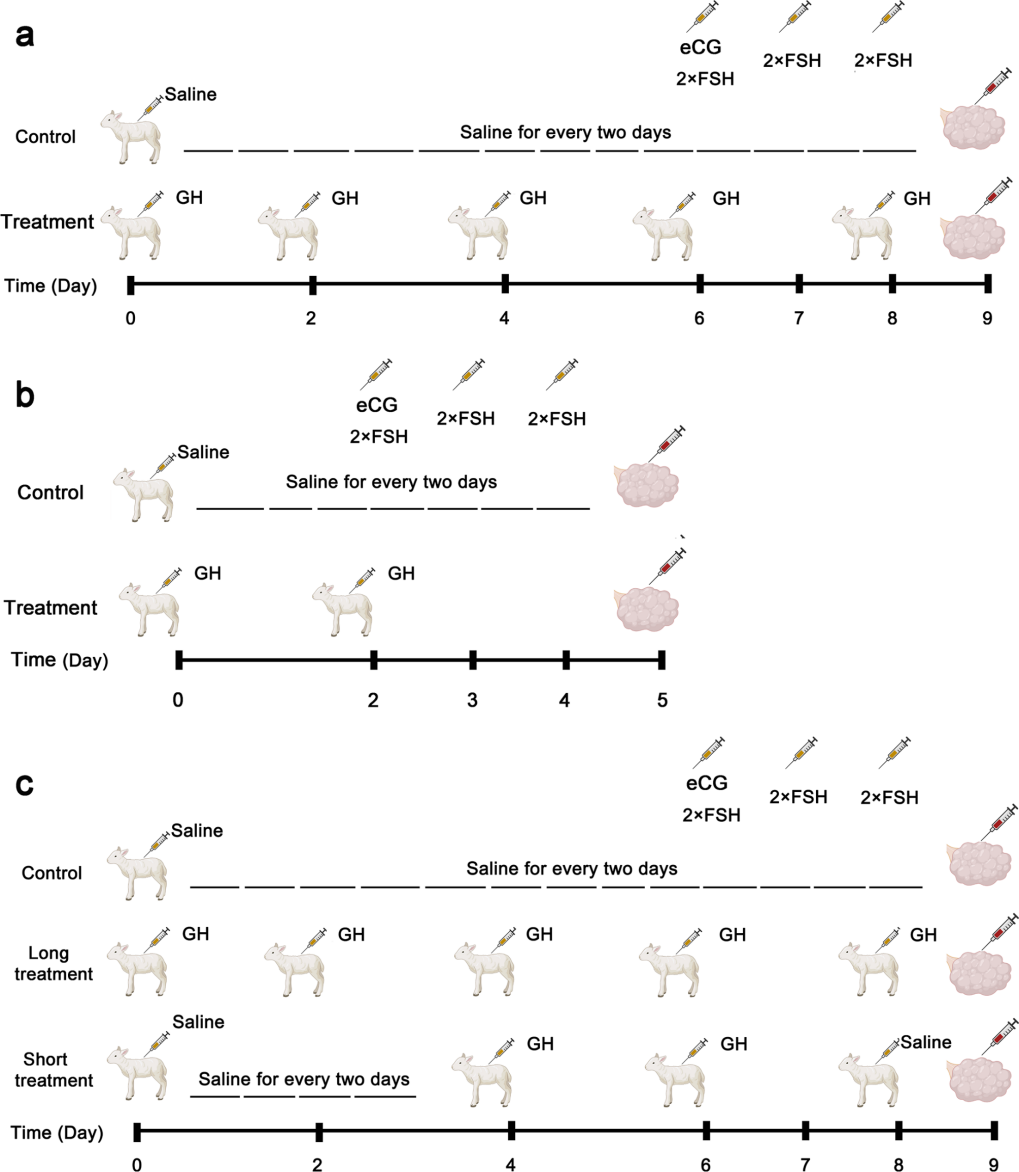
Schematic diagram illustrating the experiments for GH treatment and superovulation of lambs. **a** Long GH treatment. The lambs were treated with a total of 50 mg (high dose) or 25 mg (low dose) of GH for 5 days. **b**. Short GH treatment. The lambs were treated with a total of 50 mg (high dose) or 20 mg (low dose) of GH for 2 days. **c** Comparison of long and short GH treatment. The lambs were treated with a total of 50 mg of GH for 5 days (long treatment) or 2 days (short treatment). Saline injections were used as controls. Superovulation was induced with injection of eCG and FSH.

For GH treatment, the lambs were subcutaneously injected with a recombinant ovine GH that was produced in our lab as described previously [23]. As illustrated in Fig. 1, different dosages and time points of GH injections were designed to examine the effects of GH treatment on JIVET efficiency. In trial 1, a total of 21 lambs were randomly allocated to three groups, with 7 lambs in each group. The lambs used for GH treatment received a total of five injections of GH, which were administrated during the period of Day 0 to Day 8, with 10 mg (high dose) or 5 mg (low dose) for each injection (Fig. 1a, referred as long-treatment). Unfortunately, one lamb in the 50 mg group died of diarrhea during the treatment. In trial 2, a total of 18 lambs were used (n = 6 in each group), and the lambs in GH treatment groups received 2 injections of GH that were applied only at Day 0 and Day 2, with 25 mg (high dose) or 10 mg (low dose) for each injection (Fig. 1b, referred as short-treatment). In trial 3, a total of 15 lambs were randomly allocated to three groups (n = 5 in each group), and the lambs in GH treatment groups received 5 × 10 mg (long treatment) or 2 × 25 mg (short treatment) GH injections at the time points as illustrated in Fig. 1c. In all trials, in parallel with each GH injection, injection of saline solution was used as controls.

The procedure of all animal experiments was in accordance with the animal care policies of China Agricultural University and was approved by the Animal Ethics Committee at the university (protocol code: AW20701202-3-4, date of approval: 1 June, 2020).

### Oocyte in vitro maturation (IVM), in vitro fertilization (IVF) and embryo in vitro culture (IVC)

Oocytes were recovered from lambs by laparotomy approximately 12 h after the last FSH injection as described previously [9]. The cumulus-oocyte complexes (COCs) were cultured in IVM medium, TCM 199 supplemented with 20% (*v:v*) estrus sheep serum (ESS), 10 µg/mL FSH (Folltropin-V; Bioniche Inc. Belleville, Ont., Canada), 10 µg/ml luteinizing hormone (LH, Bioniche Inc), 1 µg/mL 17β-estradiol, 100 IU/mL penicillin and 100 µg/mL streptomycin, at 38.5 ℃ in a humidified atmosphere of 5% CO_2_ in air.

After 24 h of culture, the COCs were partially stripped of cumulus cells by briefly incubating in 0.2% hyaluronidase in Hepes-buffered TCM199 solution, and then placed into IVF medium, synthetic oviduct fluid (SOF) medium containing 2% ESS. Frozen-thawed sperm were layered under 600 µL IVF medium in a glass tube, and incubated for 30 min at 38.5 ℃ for swimming up. Swim-upped spermatozoa were collected from the upper fraction and added into each IVF well, with a final density of 1 × 10^5^ spermatozoa mL ^− 1^. The COCs and sperm were co-incubated for 24 h at 38.5 ℃ under 5% CO_2_ in air.

Following IVF, the presumptive zygotes were cultured in IVC medium, SOF containing 8 mg/mL fatty acid-free bovine serum albumin, 1% (*v*:*v*) essential amino acids and 2% (*v*:*v*) non-essential amino acids. Zygotes were incubated in a humidified atmosphere of 5% CO_2_, 5%O_2_, and 90% N_2_ at 38.5 °C. Cleavage and blastocyst development were recorded after 48 h and 7–9 d of IVC, respectively.

### Blood samples and hormonal assays

In trial 1 and 2, the serum was sampled from the lambs every day from Day 0 until the day of oocyte retrieval. The serum progesterone (P_4_) and estradiol (E_2_) levels were measured by radioimmunoassay (RIA) using commercial RIA kits (Beijing North Institute of Biological Technology, China). The serum insulin-like growth factor-1 (IGF-1) levels were measured by Enzyme-linked immune sorbent assay (ELISA) (IGF-1 600 ELISA, DRG International, Inc., USA).

### GC culture and treatment

The GCs derived from lamb ovaries without GH treatment were cultured in D-MEM/F12 (Gibco, Invitrogen Corporation, Grand Island, NY, USA) supplemented with 10% fetal bovine serum (FBS). To verify the effects of GH and IGF-1, the GCs were cultured in D-MEM/F12 containing 2% FBS, and relevant indicators were assayed after GH or IGF-1 treatment.

### Fluorescence staining

Immediately after retrieval from the lamb ovaries, the oocytes (at germinal vesicle stage) were stained for 20 min at 38.5℃ with 1 mM dichlorofluorescein diacetate (DCFH-DA), 10 µM 4-chloromethyl-6,8-difluoro-7-hydroxycoumarin (Cell-Tracker Blue) and 5 µM Flou-3 AM for detection of the levels of reactive oxygen species (ROS), glutathione (GSH) content, and cytosolic Ca^2+^, respectively. Oocytes stained with 200 nM Mito-tracker (Red) (Beyotime Institute of Biotechnology, China) or 10 µmol/L MitoProbe JC-1 (Beyotime Institute of Biotechnology) for detection of the mitochondrial distribution or mitochondrial membrane potential (MMP), respectively. The fluorescence was measured under an epifluorescence microscope (DP72, Olympus, Tokyo, Japan). For cells, the GSH content was measured by ELISA (GSH ELISA Kit, Zhenke Biotechnology Co., LTD, Shanghai, China). Cells were stained with Annexin V and propidium iodide (PI) for apoptosis analysis detected by flow cytometry.

### Immunocytochemistry (ICC)

The GCs were fixed with 4% paraformaldehyde (PFA) for 30 min at 4 °C, and then incubated with 1% PBST (1% Triton X 100 dissolved in PBS) for 30 min at room temperature. The samples were then blocked with blocking solution (Beyotime, P0260) and then incubated with the primary antibodies (Table S1) in blocking solution overnight at 4 °C, followed by incubation with secondary antibodies at 37 °C for 1.5 h. The nuclei of cells were stained with Hoechst33342 (Beyotime, C1022). Fluorescent images were captured using a fluorescent microscope (Olympus, BX51, Japan). The relative fluorescence intensity per unit area was determined using Image J software (National Institutes of Health, USA).

### Immunohistochemical staining

Ovaries were removed from lambs and fixed in 4% paraformaldehyde. After fixation, pieces of ovaries were embedded in paraffin, sectioned to approximately 5 μm, and mounted on glass slides. The ovarian sections were deparaffinized in xylene, rehydrated, and retrieved by microwave heating with a buffer of Citrate Antigen Retrieval Solution (Beyotime, P0081) for 20 min. Endogenous peroxidase activity was quenched by incubation with 3% H_2_O_2_ for 10 min. After blocking in 1% BSA for 1 h, sections were immunostained with anti-IGF-1 primary antibody (Table S1) and biotin-labeled secondary antibody.

### Quantitative real-time-PCR (qRT-PCR)

Total RNA was extracted from cells using the TRNzol Reagent (Tiangen Biotech, Beijing, China) and was reverse transcribed using the FastQuant RT Kit (Tiangen Biotech) following the standard protocol. The qPCR reaction was performed using SuperReal PreMix Kit (Tiangen Biotech) on an ABI 7500 system (Applied Biosystems). The cycle threshold (CT) value of genes was obtained from three replicates. The expression level was normalized to that of the internal gene *GAPDH*. The primers used are presented in Table S2.

### Western blotting

Lamb GCs cultured in 35-mm dishes were lysed with RIPA (Cwbio, Jiangsu, China) containing 1 mM PMSF and phosphatase inhibitor (Beyotime). Cell lysates were separated by 12% SDS polyacrylamide gel electrophoresis (SDS-PAGE) and transferred onto nitrocellulose membranes. The membranes were blocked with blocking solution (Beyotime) and incubated with primary antibodies (Table S1) overnight at 4℃, followed by incubation with horseradish peroxidase (HRP)-conjugated secondary antibodies for 1.5 h at room temperature. The β-actin protein was used as a loading reference. The membranes were treated with super ECL Plus and the western blotting signals were detected by Tanon 5200 fully automatic chemiluminescence system. The intensities of blotting signals were quantified by outlining the relevant bands on the film with Image J software.

### Transcriptomic analysis of GCs

After COCs were picked up from the aspiration of ovarian follicles of lambs, the remaining GCs were collected and the total RNA was extracted using TRIzol method (DP424, Tiangen, Beijing) according to the manufacturer’s instructions. The RNA was quantified using Agilent 2100 Bioanalyzer (Agilent Technologies, CA, USA), and the quality and integrity were assessed by NanoDrop spectrophotometer (IMPLEN, CA, USA). RNA-seq was performed with the PE150 sequencing strategy using an Illumina second-generation high-throughput sequencing platform. The adaptor sequences and low-quality sequence reads were removed from the raw data through In-House perl scripts. The clean reads were mapped to the reference genome sequence (https://sheephapmap.org/news/OARv2p0) by STAR. Only reads with a perfect match or one mismatch were further analyzed and annotated based on the reference genome.

For analysis of the differentially expressed genes (DEGs), HTSeq v 0.5.4 p3 was used to count the reads numbers mapped to each gene, and the gene expression levels were estimated by fragments per kilobase of transcript per million fragments mapped (FPKM). Subsequently, differential expression analysis of two conditions/groups was performed using the DESeq R package (1.10.1). Transcripts with P-value < 0.05 and |log2FC| > 1 were considered differentially expressed. GO enrichment analysis and KEGG pathway enrichment analysis of the DEGs were implemented by the GOseq R packages and KOBAS software, respectively.

### Proteome of ovaries

Ovaries were removed from lambs, rinsed with saline and stored at -80℃. The extraction of total proteins referred to the method of Yang et al. [24]. A BCA Protein Assay Kit (Beijing Solarbio Science & Technology, Beijing, China) was used to measure protein concentration, and contamination and degradation of protein extracts were checked by SDS-PAGE. The proteins (100 µg each) were digested using the FASP method [25]. High-performance liquid phase separation was carried out by Thermo UltiMate 3000 UHPLC. The peptides were separated by liquid phase, ionized by a nanoESI source, and then transferred to a tandem mass spectrometer Q-Exactive HF (Thermo Fisher Scientific, San Jose, CA) for DIA mode detection.

The obaDIA software was used for MS data analysis via the chromatogram library (obaDIA: one-step biological analysis pipeline for data-independent acquisition and other quantitative proteomics data). A peptide or protein FDR < 1% was set as the protein identification threshold. The differentially expressed proteins (DEPs) were identified using mapDIA. Proteins with a minimum of 2-fold change (|log2(FC)|>1) and false discovery rate (FDR) < 0.05 were identified as DEPs. The GOseq R package (v1.12) and KEGG Orthology Based Annotation System software (KOBAS, v2.0) were used to determine the DEPs in GO and KEGG pathways, respectively. A corrected *P*-value < 0.05 was considered as the significant cutoff value.

### FF metabolite profiles

FF was collected by aspiration of follicles of lamb ovaries and centrifuged for removing any cell debris. Metabolite samples were prepared following the method of Want et al. [26]. LC-MS/MS metabolites were measured by SCIEX UPLC system (Exion LC, SCIEX, Concord, New Hampshire) and quadrupole-time-of-flight mass spectrometer (QTOF MS; Triple TOF 5600+, SCIEX, USA). Metabolites were identified by automatically comparing retention times, ion signatures, and tandem mass spectrometry fragmentation patterns. To refine the analysis of the results, we obtained the first principal component of variable importance in the projection (VIP). Differential metabolites (DMs) were screened by VIP ≥ 1 and |Log2(FC)|>1. KEGG (http://www.kegg.jp) and MetaboAnalyst databases (http://www.metaboanalyst.ca/) were used to search for metabolite pathways.

### Statistical analyses

Statistical analysis was performed using the SPSS 24 Software. The numbers of recovered oocytes among groups were compared by Mann-Whitney U-test. The levels of serum hormones (IGF-1, P_4_, E_2_), the rates of cleavage and blastocysts, and the data in cellular experiments were statistically analyzed by Student’s two-tailed t-test. The fluorescence intensities of stained oocytes were analyzed by One-way ANOVA. All data were expressed as mean value ± standard error of the mean (SEM). Differences were considered significant at three levels (*P<0.05; **P<0.01; ***P<0.001).

## Results

### Effects of GH treatment on development of lamb oocytes

#### Long-GH treatment

**Fig. 2:**
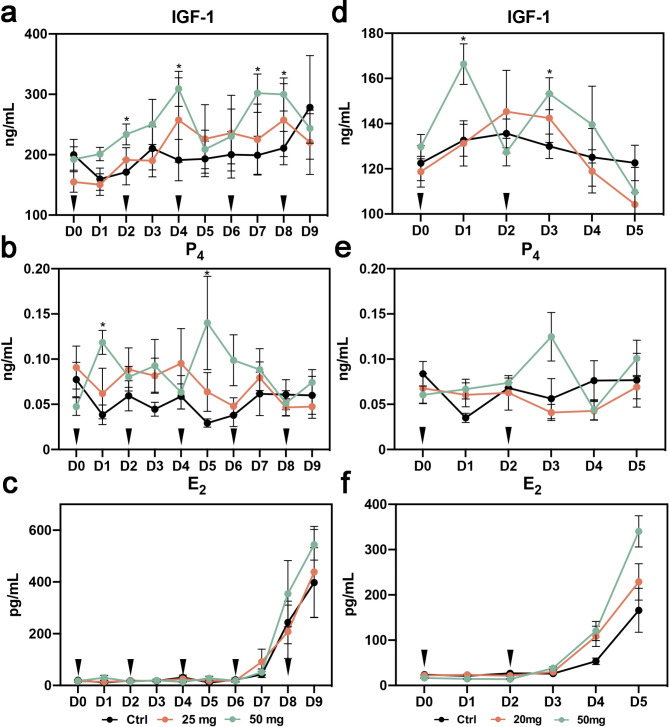
Serum hormone levels in lambs during the period of treatment. **a-c** The changes of serum IGF-1, P_4_ and E_2_ levels in lambs in trial (1) **d-f** The changes of serum IGF-1, P_4_ and E_2_ levels in lambs in trial (2) *, *P* < 0.05. Arrow heads indicate the day of GH injection

Trial 1 examined the effects of two dosages in long-GH treatment on the lamb oocyte development. Table 1 shows the response of lambs to superovulation and the oocyte recovery from individual lambs. Although there was no significant difference in average oocyte recovery among three groups of lambs, 50 mg GH treatment (5 × 10 mg GH) had a trend of increasing the number of responding lambs and the average number of oocytes recovered from per lamb. After fertilization of the recovered oocytes, no significant differences in cleavage and blastocyst rates were found between GH treatment and control groups, but significantly higher percentage of fast-developing blastocysts (appeared in D7 and D8) was observed in the 50 mg GH group (34.2%) compared to the 25 mg GH group (22.6%) and control group (22.1%) (Table 2). Assays for serum hormones showed that 50 mg GH treatment significantly increased the circulating IGF-1 concentrations in lambs (Fig. 2a). The levels of serum P_4_ and E_2_ were also increased to a degree in the 50 GH treatment group, albeit not significantly different from the control (Fig. 2b and c). These results suggest that long treatment of lambs with 50 mg GH had a beneficial role on lamb oocyte development.

**Table 1:** Number of oocytes recovered from lambs in trial 1

Control		25 mg GH		50 mg GH	
Serial number of lambs	No. of recovered oocytes	Serial number of lambs	No. of recovered oocytes	Serial number of lambs	No. of recovered oocytes
#1	100	#8	77	#15	34
#2	63	#9	35	#16	260
#3	No response	#10	No response	#17	76
#4	67	#11	30	#18	35
#5	No response	#12	28	#19	184
#6	253	#13	139	#20	168
#7	66	#14	145		
Total	549		454		757
Average oocyte number (all lambs)	78.4 ± 32.3		64.9 ± 21.7		126.2 ± 37.6
Average oocyte number (responding lambs)	109.8 ± 36.4		75.7 ± 22.2		126.2 ± 37.6

No response: The number of follicles on the ovarian surface ≤ 3

**Table 2:** Developmental ability of oocytes in trial 1

Groups	No. of oocytes inseminated	No. of cleaved (Mean ± SEM, %)^a^	Blastocysts			
			No. of D7 + D8 blastocysts^b^	Rate of D7 + D8 blastocysts (Mean ± SEM,%)^c^	Total^d^	Rate of total blastocysts (Mean ± SEM,%)^e^
Control	442	305 (66.9 ± 9.4)	72	22.1 ± 3.9^f^	107	31.3 ± 9.1
25 mg GH	365	289 (66.3 ± 11.9)	80	22.6 ± 5.7^f^	103	30.2 ± 5.1
50 mg GH	642	421 (60.9 ± 6.1)	145	34.2 ± 3.1^ g^	163	36.8 ± 4.0

a: Percentage of cleaved = no. of cleaved/no. of oocytes inseminated

b: Total no. of blastocysts developed at day 7 (D7) and day 8 (D8) after cleavage

c: Total no. of blastocysts in D7 + D8/no. of cleaved

d: Total no. of blastocysts developed from day 7 to day 9

e: Total no. of blastocysts/no. of cleaved

Values with different letters (f and g) within the same column are significantly different (P < 0.05)

#### Short-GH treatment

Results from trial 1 suggested a positive role of GH applied as 5 × 10 mg. In trial 2, the efficacy of short-GH treatment of lambs was determined, which 2 possible parameters, 50 mg GH in total or 10 mg GH in each injection, were taken into consideration. As shown in Tables 3, 50 mg GH treatment (2 × 25 mg GH) significantly increased the average number of recovered oocytes as compared to the control and 20 mg groups (2 × 10 mg GH). Notably, in this trial, only 2 of 6 lambs in the control group responded to the FSH stimulation, while all of lambs in 50 mg GH group had responses to the FSH stimulation. However, no significant differences were found in cleavage and blastocyst development among three groups (Table 4). Similar to that in trial 1, injection of 50 mg GH resulted in significant rise of serum IGF-1 concentrations in lambs (Fig. 2d). The levels of P_4_ and E_2_ were also slightly, albeit not significantly, elevated in 50 mg GH treatment (Fig. 2e and f). These results suggest that short treatment of lambs with 50 mg GH also had a potential of improving the JIVET efficiency.

**Table 3:** Number of oocytes recovered from lambs in trial 2

Control		20 mg GH		50 mg GH	
Serial number of lambs	No. of recovered oocytes	Serial number of lambs	No. of recovered oocytes	Serial number of lambs	No. of recovered oocytes
#1	No response	#7	65	#13	47
#2	No response	#8	No response	#14	133
#3	No response	#9	No response	#15	54
#4	No response	#10	121	#16	57
#5	101	#11	149	#17	51
#6	15	#12	23	#18	19
Total	116		358		361
Average oocyte number (all lambs)	19.3 ± 16.5^a^		59.7 ± 25.9^a^		60.2 ± 15.6^b^
Average oocyte number (responding lambs)	58.0 ± 43.0		89.5 ± 28.2		60.2 ± 15.6

No response: The number of follicles on the ovarian surface ≤ 3

Values with different letters (a and b) within the same row are significantly different (P < 0.05)

**Table 4:** Developmental ability of oocytes in trial 2

Groups	No. of oocytes inseminated	No. of cleaved (Mean ± SEM, %)^a^	Blastocysts			
			No. of D7 + D8 blastocysts^b^	Rate of D7 + D8 blastocysts (Mean ± SEM,%)^c^	Total^d^	Rate of total blastocysts (Mean ± SEM,%)^e^
Control	100	79 (77.3 ± 2.3)	6	9.1 ± 1.9	12	27.9 ± 16.5
20 mg GH	306	234 (65.5 ± 15.2)	30	10.4 ± 5.1	42	14.4 ± 7.7
50 mg GH	311	249 (76.7 ± 4.1)	38	14.3 ± 2.6	49	16.4 ± 3.8

a: Percentage of cleaved = no. of cleaved/no. of oocytes inseminated

b: Total no. of blastocysts developed at day 7 (D7) and day 8 (D8) after cleavage

c: Total no. of blastocysts in D7 + D8/no. of cleaved

d: Total no. of blastocysts developed from day 7 to day 9

e: Total no. of blastocysts/no. of cleaved

#### Comparison of long- and short- GH treatment

The above results indicated that 50 mg GH could achieve a higher efficiency in terms of lamb response and embryo yield in either long or short treatment. In trial 3, we compared the effects of long and short treatment, both of which applied 50 mg GH in total. In this trial, although all three groups of lambs, including the control group, had satisfactory responses to FSH stimulation (Table 5), the blastocyst development in both GH treatment groups was significantly enhanced compared with that of the control group (Table 6). This result supports again that 50 mg GH treatment could improve the developmental ability of lamb oocytes.

**Table 5:** Number of oocytes recovered from lambs in trial 3

Control		Short GH treatment		Long GH treatment	
Serial number of lambs	No. of recovered oocytes	Serial number of lambs	No. of recovered oocytes	Serial number of lambs	No. of recovered oocytes
#1	131	#6	74	#11	175
#2	9	#7	54	#12	58
#3	120	#8	166	#13	149
#4	185	#9	80	#14	71
#5	99	#10	65	#15	93
Total	544		439		546
Average oocyte number	108.8 ± 28.7		87.8 ± 20.1		109.2 ± 22.6

**Table 6:** Developmental ability of oocytes in trial 3

Groups	No. of oocytes inseminated	No. of cleaved (Mean ± SEM, %)^a^	Blastocysts			
			No. of D7 + D8 blastocysts^b^	Rate of D7 + D8 blastocysts (Mean ± SEM,%)^c^	Total^d^	Rate of total blastocysts (Mean ± SEM,%)^e^
Control	453	269 (61.7 ± 4.6)	84	28.9 ± 2.7^f^	90	30.9 ± 3.2^f^
Short treatment	203	114 (54.9 ± 8.9)	54	44.8 ± 4.2^ g^	55	46.2 ± 4.8^ g^
Long treatment	389	244 (65.9 ± 8.8)	104	40.7 ± 3.4^ g^	111	43.6 ± 3.5^ g^

a: Percentage of cleaved = no. of cleaved/no. of oocytes inseminated

b: Total no. of blastocysts developed at day 7 (D7) and day 8 (D8) after cleavage

c: Total no. of blastocysts in D7 + D8/no. of cleaved

d: Total no. of blastocysts developed from day 7 to day 9

e: Total no. of blastocysts/no. of cleaved

Values with different letters (f and g) within the same column are significantly different (P < 0.05)

#### Cytoplasmic evaluation of oocytes

**Fig. 3:**
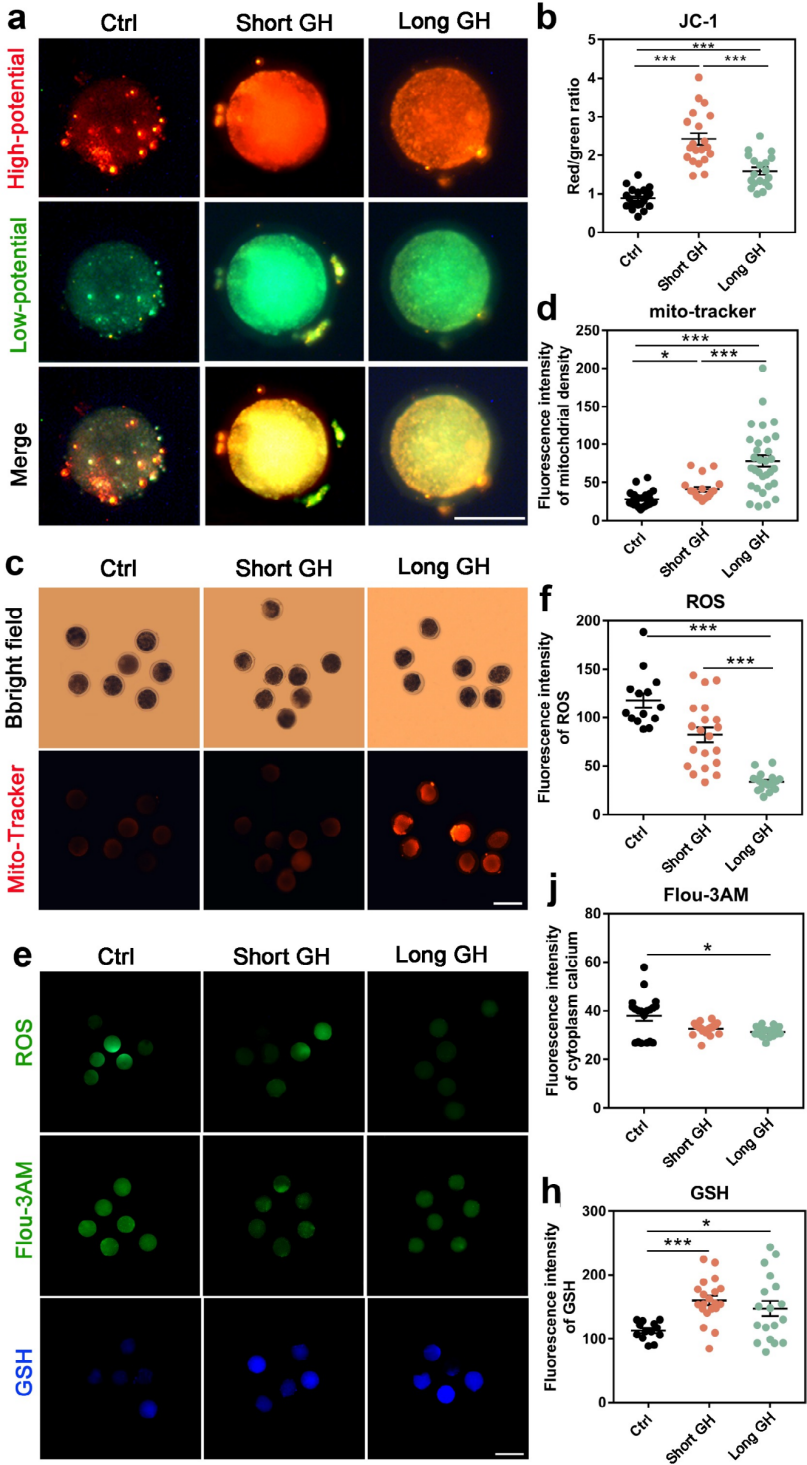
Detection of the relative indexes of lamb oocyte quality in trial 3. **a** The representative images of staining for MMP in lamb oocytes (scale bar = 50 μm), Red indicates high Δφm, while green indicates low Δφm. **b** The quantification of MMP levels detected by fluorescence intensities. **c** The representative images of mitochondrial masses staining (Mito-Tracker) in lamb oocytes (scale bar = 100 μm). **d** The quantification of mitochondrial masses detected by fluorescence intensities. **e** The representative images of staining for ROS, cytosolic Ca^2+^ (Floµ-3AM) and GSH content in lamb oocytes from top to bottom (scale bar = 100 μm). **f-j** The quantification of levels of ROS, cytosolic Ca^2+^ and GSH content detected by fluorescence intensities. Ctrl, control; long GH, long GH treatment (for 5 days); short GH, short GH treatment (for 2 days). *P<0.05; ***P<0.001

To further understand the influence of GH treatment of lambs on oocyte quality, several aspects of oocyte cytoplasm were detected in trial 3. As shown in Fig. 3, GH treatment significantly increased the MMP (Fig. 3a, b) and the mitochondrial mass (Fig. 3c, d). The levels of ROS and cytosolic Ca^2+^ in oocytes were significantly decreased in long treatment group compared to the control group, and the GSH content was significantly higher in the two GH treatment groups than that of the control group (Fig. 3e, f-h). These results suggest that GH treatment could improve the mitochondrial function and reduce the oxidant stress (OS) of lamb oocytes.

### Transcriptome analysis of GCs

**Fig. 4:**
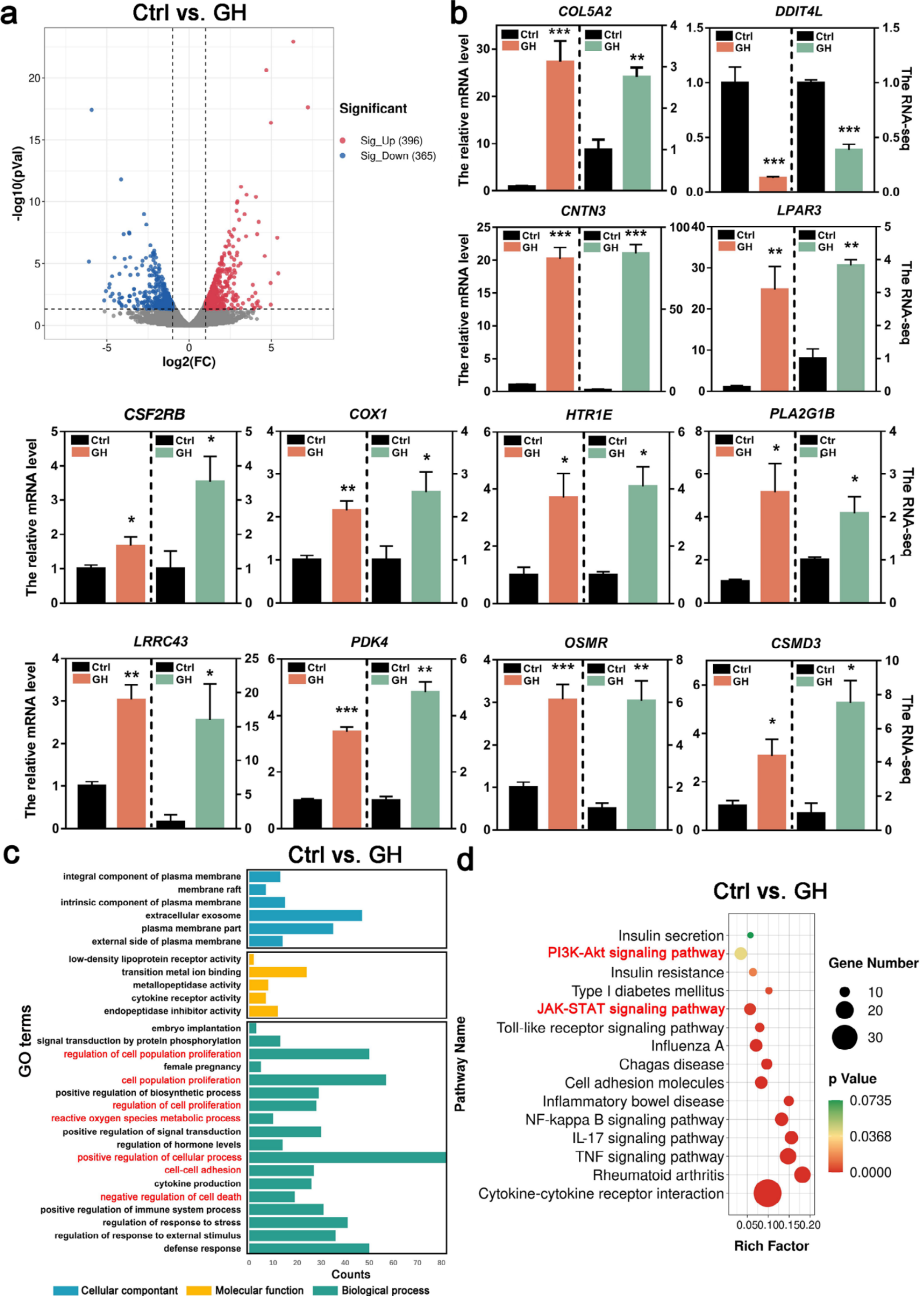
RNA-seq data shows gene expression changes between control and GH treatment groups. **a** Volcano plots show DEGs. The x-axis shows the log2(FC) in gene expression, and the y-axis shows significant statistical differences. Red, up-regulated genes; blue, down-regulated genes. **b** Verification of expression levels of DEGs by qRT-PCR. **c** GO enrichment analysis of DEGs from enrich Gene Ontology for biological processes (BP), cellular components (CC) and molecular functions (MF). **d** KEGG enrichment analyses of DEGs. The ratio of the number of DEGs to the total gene number is represented by the enrichment factor. Size of dots: number of genes; color of dots: range of p-values. *P<0.05; **P<0.01; ***P<0.001

To further explore the underlying mechanism regarding how GH treatment improved the ovarian response and the quality of lamb oocytes, we analyzed the transcriptome profiles of GCs from long-GH treatment and control by RNA sequencing (RNA-seq). A total of 761 DEGs were identified between GH-treatment and control group (Fig. 4a and Supplementary additional file 1). The RNA-seq data were further verified by qRT-PCR with randomly selected genes (Fig. 4b).

Next, we conducted GO term and KEGG enrichment analysis for the DEGs. The GO enrichment analysis revealed that many DEGs were implicated in the regulation of cell proliferation, ROS metabolic process and negative regulation of cell death (Fig. 4c). The KEGG enrichment analysis indicated that several pathways, such as the PI3K/Akt and JAK/STAT signaling pathways, were activated in GCs from GH-treated lambs (Fig. 4d). The above results suggested that GH probably had a role in promoting the GC proliferation, inhibiting the GC apoptosis and reducing OS, and GH might function through activating the PI3K/Akt signaling pathway.

### Proteome analysis of the ovaries

**Fig. 5:**
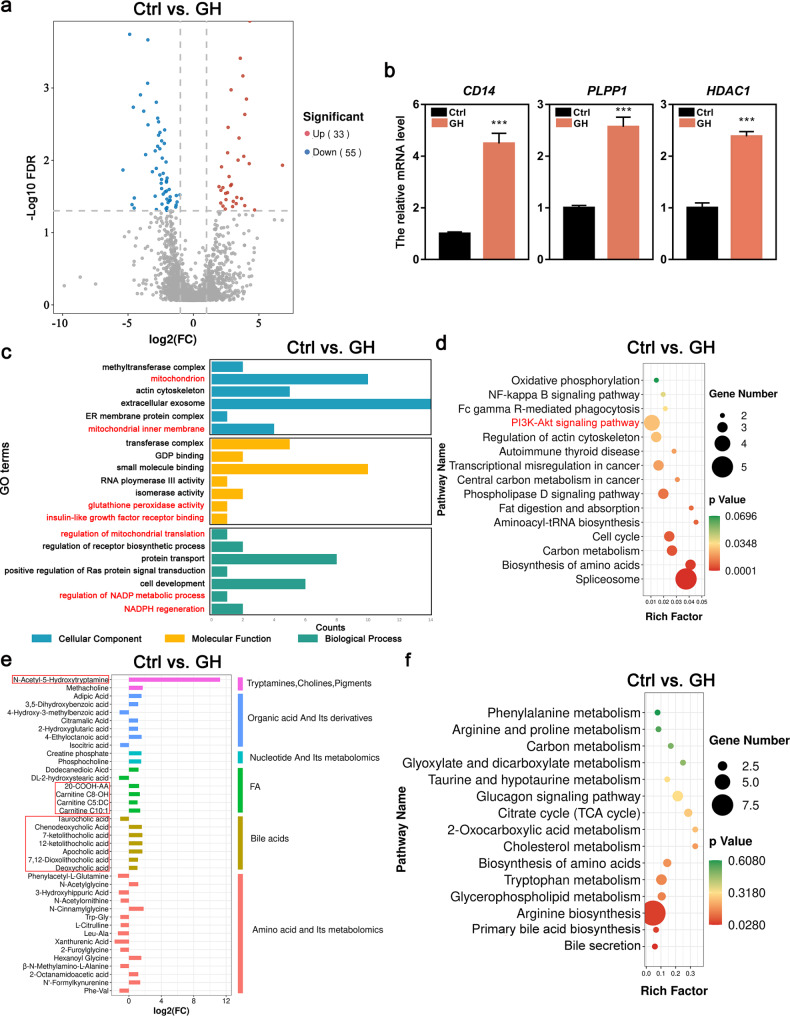
Proteome and metabolome data. **a** Volcano plots show DEPs. The x-axis shows the log2(FC) in protein expression, and the y-axis shows significant statistical differences. Red, up-regulated proteins; blue, down-regulated proteins. **b** Verification of expression levels of DEPs by qRT-PCR. **c** GO enrichment analysis of DEPs from enrich Gene Ontology for biological processes (BP), cellular components (CC) and molecular functions (MF). **d** KEGG enrichment analyses of DEPs. The ratio of the number of DEPs to the total protein number is represented by the enrichment factor. Size of dots: number of proteins; color of dots: range of p-values. **e** Classification chart of all the annotated DMs. **f** KEGG enrichment analyses of DMs. The ratio of the number of DMs to the total metabolite number is represented by the enrichment factor. Size of dots: number of metabolites; color of dots: range of p-values. ***P<0.001

When the proteome of ovaries was analyzed, a total of 88 DEPs were found to be significantly different between control and GH treatment groups (Fig. 5a and Supplementary additional file 2). Some of the DEPs were further verified by qRT-PCR with randomly selected proteins (Fig. 5b). GO term and KEGG enrichment analysis were used for exploring the possible function of DEPs. The enriched GO terms included the mitochondrion inner membrane, glutathione peroxidase activity, regulation of NADP metabolic process and NADPH regeneration and so on (Fig. 5c). Similar to the results of transcriptome KEGG enrichment, the proteome analysis also showed that the PI3K/Akt signaling pathway was enriched (Fig. 5d).

### Metabolome analysis of the follicular fluid

The metabolome of follicular fluid was analyzed to understand the microenvironment for oocyte growth and maturation. A total of 39 DMs were identified in the follicular fluid between control and GH treatment groups, and all the DMs were classified into 6 taxonomies (Fig. 5e and Supplementary additional file 3). In DMs, N-Acetyl-5-Hydroxytryptamine (NAS), the precursor of melatonin, was significantly increased in the GH treatment group compared with the control group. Meanwhile, bile acids (BAs) and carnitine in GH treatment group were significantly higher than that in the control group. The KEGG pathway enrichment analysis of the DMs showed that the enriched pathways mainly included amino acid metabolism, mitochondrial energy metabolism, lipid and cholesterol metabolism pathways (Fig. 5f).

### GH treatment improved the proliferation of lamb GCs

Based on the transcriptome data, we investigated the effect of GH on GCs cultured in vitro. We first determined the optimal GH treatment duration for GCs and found that treatment of cells with GH for 12 h significantly increased the IGF-1 mRNA expression, demonstrating the effectiveness of the treatment (Fig. S1).

**Fig. 6:**
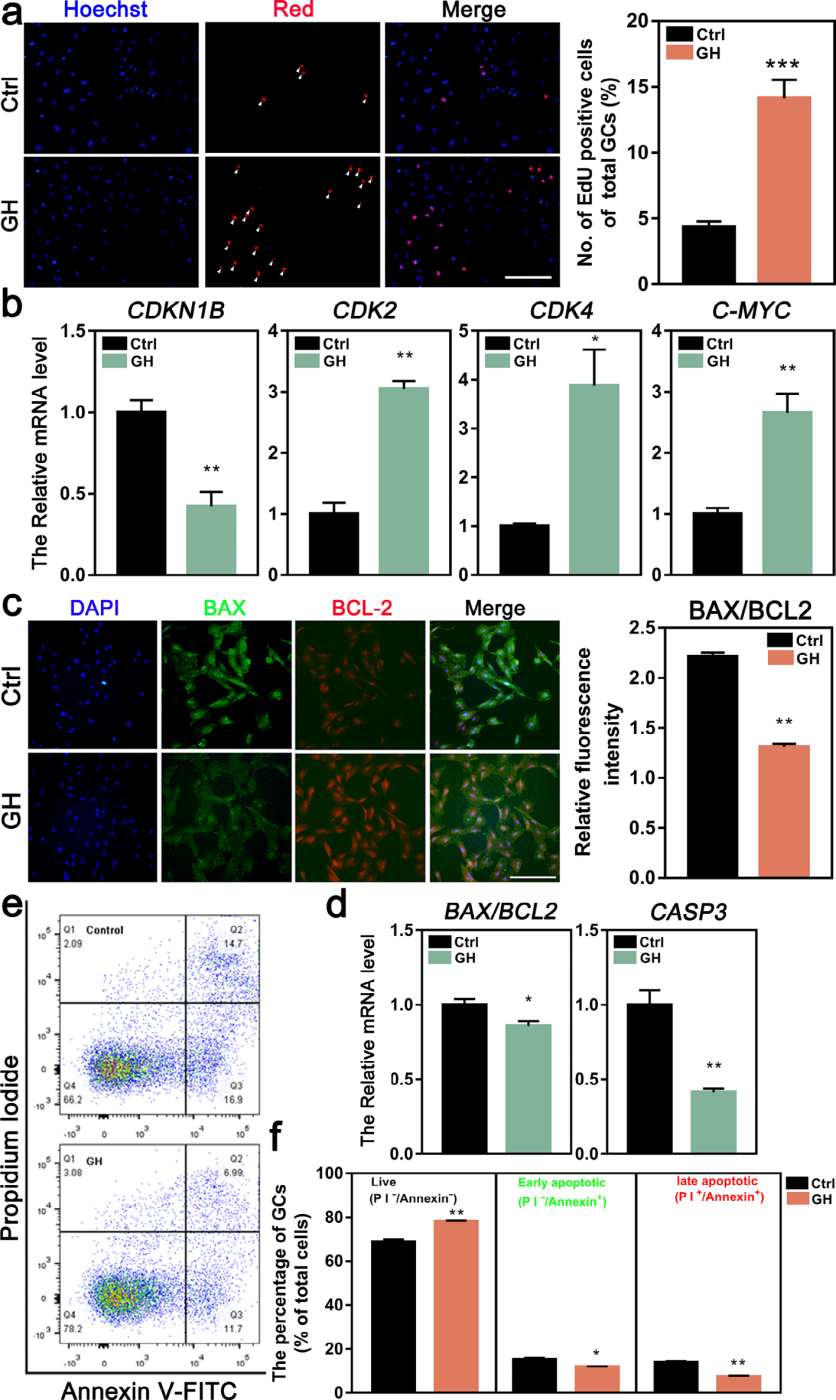
Effects of GH treatment on proliferation and apoptosis of lamb GCs. The cells were cultured in medium containing 100 µg/ml GH for 12 h and then were used for detection. **a** The representative images depicting EdU positive GCs (red) (left, scale bar = 20 μm) and the number of EdU positive cells of total GCs (right). Arrows point out EdU positive cells. **b** The relative mRNA levels of cell proliferation related genes detected by qRT-RCR. **c** Co-immunostaining of BAX (green) and BCL-2 (red) in lamb GCs (left, scale bar = 20 μm) and the quantification of BAX/BCL-2 levels in lamb GCs detected by fluorescence intensity (right). **d** The relative mRNA levels of cell apoptosis related genes detected by qRT-RCR. **e-f** The cell apoptosis was staining for PI/Annexin V and detected by flow cytometry. *P<0.05; **P<0.01; ***P<0.001

We next examined the role of GH on cell growth. EdU staining showed that the GC proliferation was significantly increased following GH treatment (Fig. 6a). Furthermore, qRT-PCR showed that GH treatment decreased the *CDKN1B* expression, and increased the expression of *CDK2*, *CDK4*, *C-MYC* (Fig. 6b). While, the protein and mRNA levels of apoptosis-related genes were significantly decreased in the GH-treated GCs compared with that in the control (Fig. 6c, d). Flow cytometry analysis by staining with Annexin V/PI revealed that the percentages of early/late apoptotic GCs were decreased by GH treatment and the numbers of viable cells were significantly increased (Fig. 6e, f). These results demonstrated that GH treatment could promote the proliferation of GCs and inhibit their apoptosis.

### GH treatment reduced the OS of lamb GCs

**Fig. 7:**
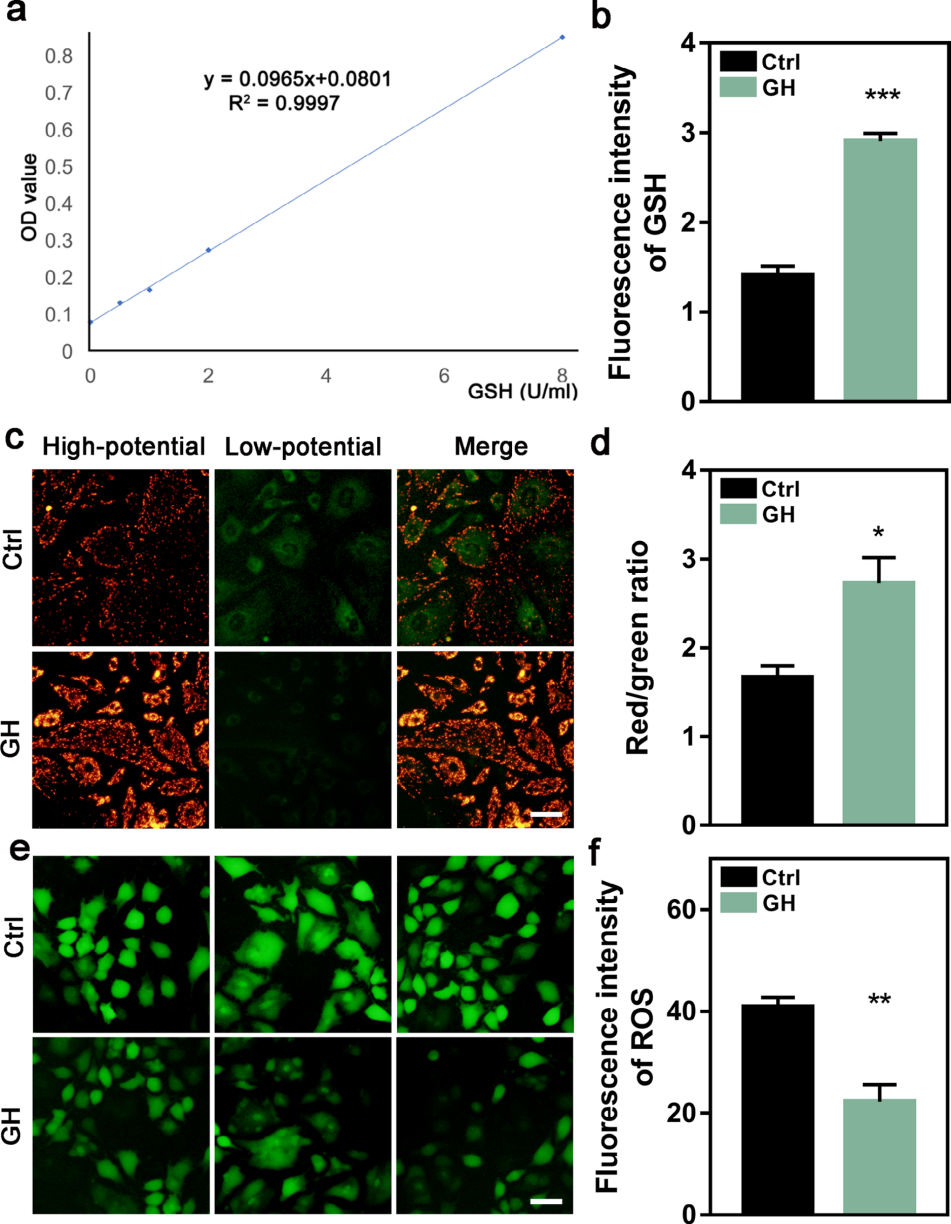
Oxidative stress levels of lamb GCs after treatment with GH. **a** The standard curve for the detection of GSH content by ELISA. **b** Quantification of GSH concentrations in GCs detected by ELISA. **c** The representative images of MMP determined in lamb GCs. **d** The quantification of MMP levels in lamb GCs detected by fluorescence intensity. **e** The representative images of ROS levels in lamb GCs. **f** The quantification of ROS levels in lamb GCs detected by fluorescence intensity. *P<0.05; **P<0.01; ***P<0.001

As transcriptome and proteome analysis had shown that GH treatment might reduce OS in lamb GCs. We tested the related index of redox state in lamb GCs treated with GH. The result showed that after GH treatment of the cells for 12 h, the GSH content (Fig. 7a, b) and MMP (Fig. 7c, d) of lamb GCs were significantly increased, while the ROS level (Fig. 7e, f) was significantly decreased. These results indicated that GH-promoted GC proliferation may be mediated by reducing OS.

### IGF-1 treatment improved the proliferation of lamb GCs

**Fig. 8:**
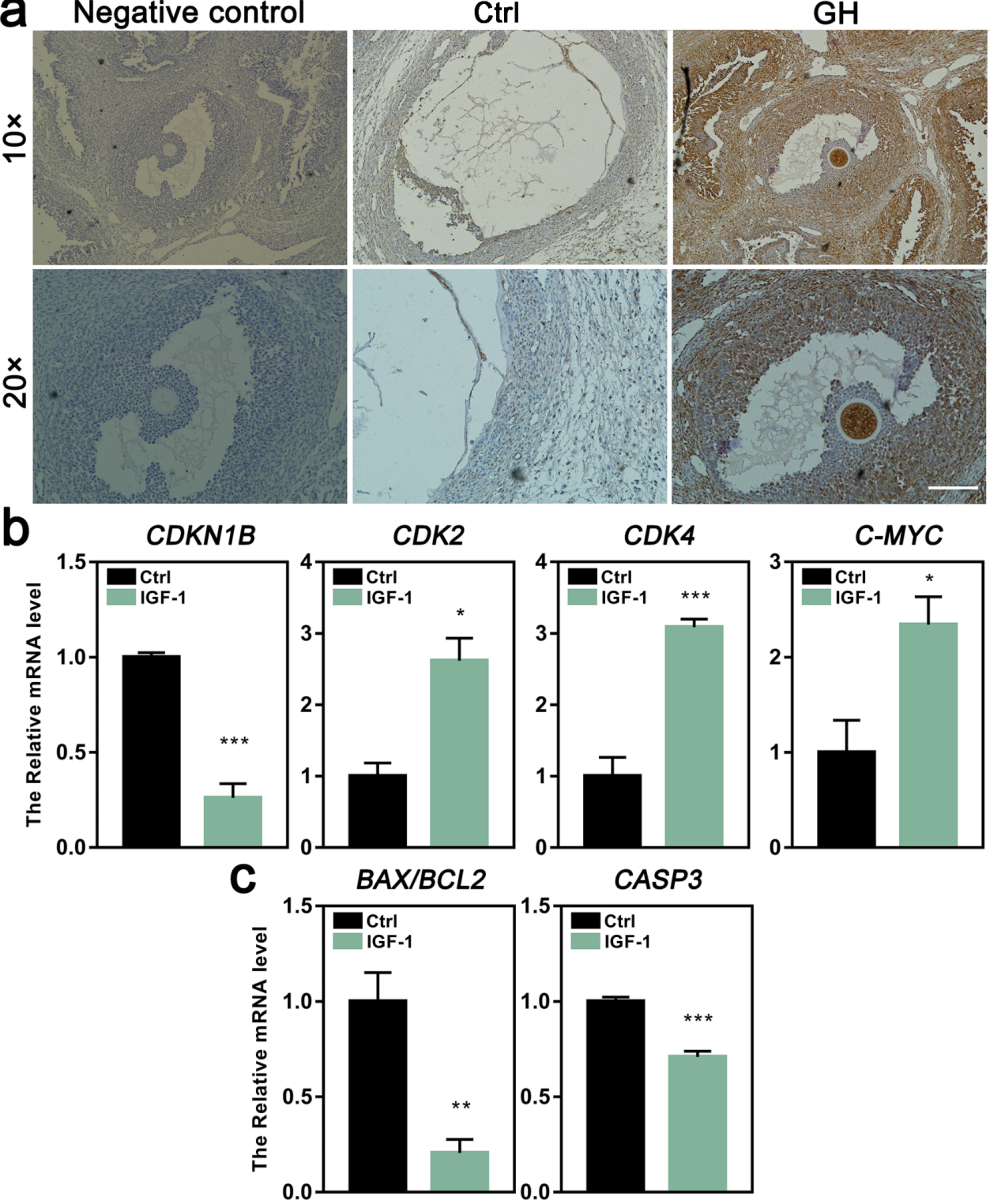
Effects of IGF-1 on lamb GCs. **a** The expression of IGF-1 in lamb ovaries detected by immunohistochemistry (scale bar = 100 μm). **b** The relative mRNA levels of cell proliferation related genes detected by qRT-RCR. **c** The relative mRNA levels of cell apoptosis related genes detected by qRT-RCR. The cultured GCs were treated with 100 ng/ml IGF-1 for 12 h and then were harvested for detection of the gene expression. *P<0.05; **P<0.01; ***P<0.001

It is well known that GH promotes the expression of liver IGF-1, which plays a critical role on cell growth. To explore the function of IGF-1 in GH-promoted GC proliferation, the expression level of IGF-1 in ovarian cells was detected by immunohistochemistry. The result showed that the expression of IGF-1 was greatly enhanced in GCs within ovaries of GH-treated lambs (Fig. 8a). We further examined the role of IGF-1 on in vitro cultured GCs, and found that IGF-1 treatment of the GCs significantly increased the expression of *CDK2*, *CDK4*, and *C-MYC*, but decreased the expression of *CDKN1B*, *BAX/BCL-2* and *CASP3* (Fig. 8b, c). These results supported a direct role of IGF-1 on GCs.

### GH functioned via the PI3K/Akt pathway

**Fig. 9:**
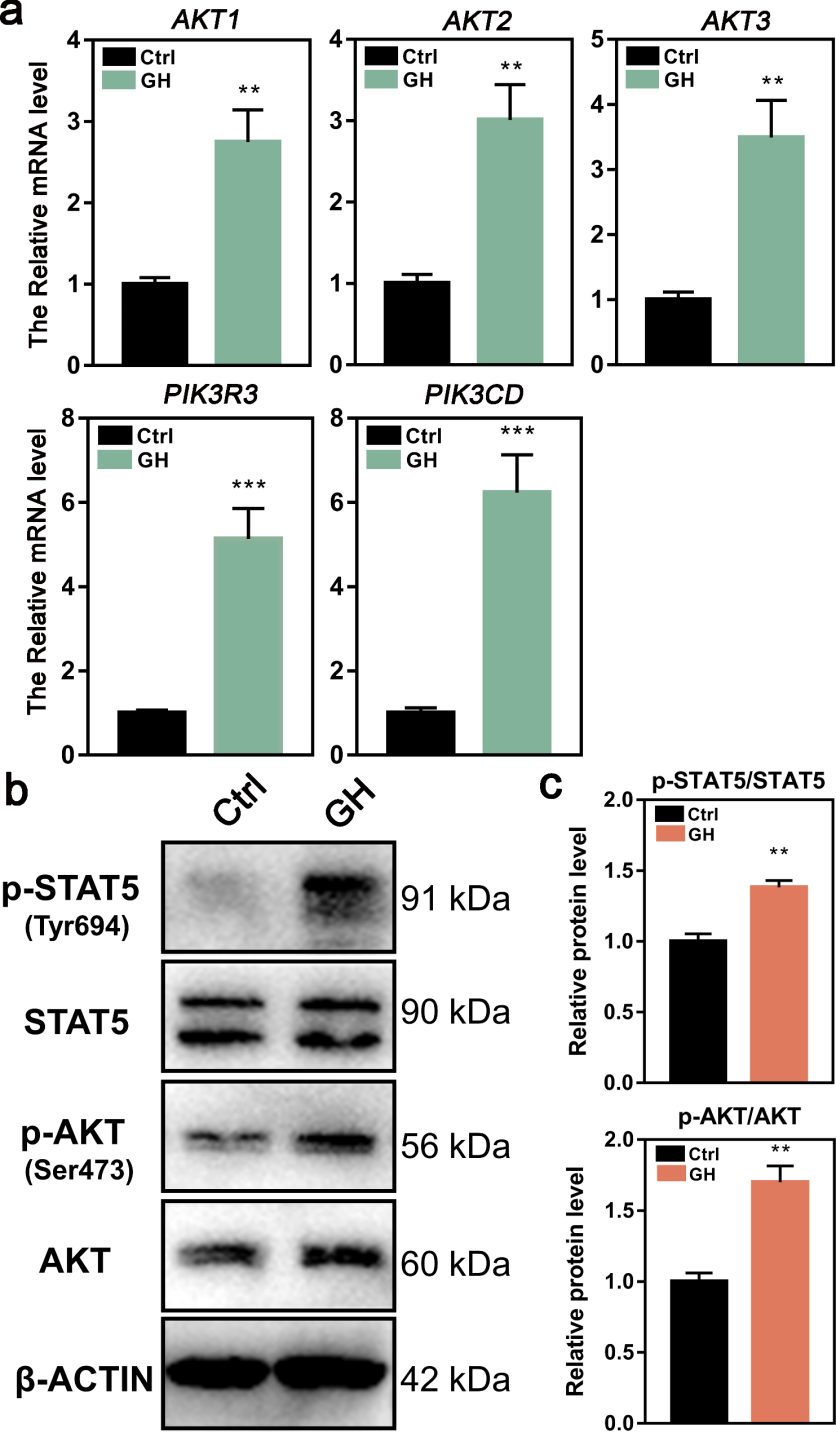
Effects of GH treatment the PI3K/Akt signaling pathway in cultured lamb GCs. **a** The relative mRNA levels of *AKT1*, *AKT2*, *AKT3*, *PIK3R3* and *PIK3CD* detected by qRT-RCR. **b** The protein expression levels of p-STAT5, STAT5, p-AKT and AKT detected by western blotting. **c** The quantification of p-STAT5/STAT5 and p-AKT/AKT levels detected by gray scanning. **P<0.01; ***P<0.001

The KEGG pathway analysis of transcriptome and proteome indicated that GH might promote lamb GC proliferation through the PI3K/ Akt signaling pathway. In addition, the JAK/STAT signaling pathway, the classical pathway that GH stimulates IGF-1 production, was enriched in transcriptome KEGG analysis. To investigate the regulating mechanism of the PI3K/Akt signaling pathway in lamb GCs treated with GH, we detected the expression of related genes in the PI3K/Akt pathway. The qRT-PCR results showed that the expression of *AKT1, AKT2, AKT3, PIK3R3* and *PIK3CD* was significantly increased in GH-treated GCs (Fig. 9a). Western blotting confirmed that the p-Akt (Ser473)/Akt ratio and p-STAT5 (Tyr694)/STAT5 ratio were significantly increased after GH treatment (Fig. 9b, c). Therefore, the PI3K/ Akt and JAK/STAT signaling pathways had been activated by GH treatment.

## Discussion

In the present study, we have explored the possibility of GH treatment on improvement of the JIVET efficiency. We showed that treatment of lambs with GH could improve the responsiveness of lambs to FSH stimulation, increase the number of recovered oocytes and enhance the development capacity of oocytes.

Many previous studies have suggested that GH could improve the reproductive performance in mammals [27, 28]. In superovulated ewes, although the regimen and dosage were different, GH treatment allowed the enhancement of the pool of gonadotrophin-responsive follicles present in the ovaries [29], increased the rates of fertilization and blastocyst development [19], and enhanced the proportion of transferable embryos [17]. In estrus-synchronized ewes, giving 50 mg GH at the start of synchronization treatment and at the time of artificial insemination increased the proportion of multiple births and prolificacy [20, 21]. Similar effects of GH were also found in cattle; treatment of cows with 500 mg GH at the time of estrus and 10 d later improved the conception rate [30]. However, it remains unknown whether GH treatment can benefit the development of prepubertal follicles and oocytes. In the present study, we demonstrated that administration of GH in lambs had a potential of promoting the development of follicles, as reflected by increasing oocyte and embryo yield in the JIVET program.

As mentioned above, one or two doses of GH are usually given in ewes or cows in estrus synchronization or superovulation [19, 21, 30]. However, when GH is applied in human assisted reproduction, the patients usually receive GH injection every day until ovulation, no matter in long-term or short-term procedures [31–34]. In the present study, we found that long-term GH treatment was more effective than short-term treatment when applied to improve the development of lamb follicles and oocytes, even though the short-term GH treatment also had a positive role. A major problem regarding in vitro embryo production from prepubertal animals is the high variation in ovarian response to FSH stimulation and oocyte development between individuals [35, 36]. This is also confirmed by our study. Lambs in the control group were quite variable in superovulation, as shown that a proportion of lambs hardly responded to FSH stimulation. In contrast, GH treatment, especially for the long-term treatment with 50 mg GH, was constantly effective in promoting all of treated lambs to respond to superovulation. Interestingly, GH treatment seemed not to increase the average oocyte recovery if compared with the oocyte recovery from the responding lambs in the control group. This may imply that the “inactive” ovaries were more likely dependent on the action of GH to become FSH-responsive, but the underlying mechanism requires further investigation.

We also found that GH treatment improved the development ability of lamb oocytes. Embryos derived from GH-treated lambs, especially in the 50 mg GH treatment group, had more potential to develop into high-quality blastocysts (e.g., blastocysts formation at day7 and day8). This suggests that GH had exerted a beneficial role on the growing oocytes within antral follicles. Several previous studies have described the positive effects of GH on oocyte quality. In humans, GH exerts a direct action, enabling the improvement of oocyte quality, via upregulation of its own receptors and enhancement of mitochondrial activity [37]. In mice, GH treatment of aged mice before standard ovarian stimulation could enhance the mitochondrial function of oocytes [38]. Similar to these observations, we found that the mitochondrial function of lamb oocytes could be improved by GH treatment, as evidenced by increased MMP and mitochondrial mass. It is known that mitochondrial dysfunction is a cause of ROS accumulation and OS generation [39]. In our study, GH treatment decreased the levels of ROS, and increased the GSH content in lamb oocytes, suggesting that the resistance to OS might have been enhanced in the oocytes. We also observed a decrease of cytosolic Ca^2+^ in oocytes from GH-treated lambs, which would facilitate modulating the Ca^2+^ level in a physiological range [40]. These observations support that the cytoplasmic maturation of oocytes had been improved by GH treatment of the animals.

To further explore the mechanism that GH treatment improves the ovarian responsiveness and promotes the follicle and oocyte development in lambs, we analyzed the GC transcriptome and ovarian proteome. The transcriptome GO-term analysis suggested that GH treatment probably improved the GC survival and growth, as lots of DEGs were enriched in regulation of cell proliferation, positive regulation of cellular process and negative regulation of cell death. Previous studies on transcriptome and proteome of ovarian cells have suggested that many DEGs or DEPs found between adult ewes and prepubertal lambs were involved in cell proliferation and apoptosis [8, 41, 42]. We also found that the ROS metabolism term enriched in transcriptome interrelates well with the results in proteomic analysis, such as mitochondrial and NADP metabolism. Importantly, the PI3K/Akt signaling pathway was enriched in both transcriptome and proteome KEGG analyses.

Based on the above results, we treated the lamb GCs with GH in vitro. We showed that GH treatment could promote the proliferation of GCs probably by increasing the resistance to OS and improving the mitochondrial function. We also confirmed in cellular experiments that GH exerted a role on GCs by activating the PI3K/Akt signaling pathway. It was reported that GH could activate the PI3K/Akt signaling pathway to alleviate GC apoptosis in PCOS patients [43]. Taken together the omics analysis with cellular validation reveal that GH treatment had altered the functions of ovarian cells in several aspects, particularly in enhancing the cell proliferation and reducing the cell apoptosis, which would consequently promote the follicle and oocyte development.

We noticed an increase of IGF-1 levels in serum and GCs in GH-treated lambs, supporting the function of GH in promoting the synthesis of IGF-1 in vivo [17, 21]. We found that IGF-1 indeed regulated the expression of genes related to cell proliferation and apoptosis in GCs cultured in vitro. The above analysis suggests that GH may directly affect the ovaries and GCs or be mediated by IGF-1. In this respect, the actions of GH and IGF1 in ovarian folliculogenesis have been well documented [28].

The influence of GH on ovaries was also reflected by alterations in follicular fluid metabolome. Follicular fluid, composed of secretions from the GCs and blood, provides a microenvironment for oocyte development and plays an essential role in follicular growth, oocyte maturation and ovulation [44–46]. Difference in metabolomic composition of follicular fluid was found between adult and prepubertal goats [47]. In this study, we observed that NAS is the most increased molecule in FF from GH-treated lambs. NAS has antioxidant and anti-apoptotic roles in OS-induced neurotoxicity by activating the TrkB/CREB/BDNF pathway and promoting the expression of antioxidant enzymes [48]. This may enhance the resistance to high OS in lamb follicles, as one-month-old lambs have a low abundance of superoxide dismutase (SOD) after superovulation [49]. In addition, the metabolism of amino acid, carnitine and BAs was very active in FF from the GH treatment group. Among them, carnitine, as a substance involved in fatty acid metabolism, may play an important role in the energy production of granulosa cells and oocytes [50]. Several studies have suggested that BAs in bovine and human FF may promote the development of oocytes [51–54]. Therefore, GH treatment had altered the FF metabolism that may benefit the development of follicles and oocytes. The real action of these metabolites needs to be further determined.

## Conclusions

In this study, we showed that GH treatment of lambs could potentially improve the responsiveness of lamb ovaries to FSH superstimulation, increase the number of recovered oocytes and enhance the developmental capacity of oocytes. The underlying mechanism was further explored by analysis of GC transcriptome, ovarian proteome and FF metabolome. We demonstrate that GH treatment could promote the GC proliferation by reducing OS through PI3K/Akt signaling pathway. These results suggest that GH treatment can affect the ovarian activity that benefits the development of follicles and oocytes. Our study provides a new strategy of improving the lamb superovulation and JIVET efficiency.

## Electronic supplementary material

Below is the link to the electronic supplementary material.

**Addition file 1**. DEGs in GC transcriptome between control and GH treatment groups.

**Additional file 2**. DEPs in ovarian proteome between control and GH treatment groups.

**Additional file 3**. DMs in follicular fluid metabolome between control and GH treatment groups.

**Additional file 4. Fig. S1**. Optimize the time for GH treatment of GCs; **Table S1**. The information of the primary antibodies; **Table S2**. Primers for quantitative real-time PCR.

## Data Availability

The datasets supporting the conclusions of this article are available from the corresponding author upon reasonable request.

## Abbreviations

GH: Growth hormone
FSH: Follicle-stimulating hormone
GC: Granulosa cell
IVP: In vitro production
JIVET: Juvenile in vitro embryo transfer
GHR: Growth hormone receptor
FF: Follicular fluid
eCG: Equine chorionic gonadotropin
IVM: In vitro maturation
IVF: In vitro fertilization
IVC: In vitro culture
COCs: Cumulus-oocyte complexes
ESS: Estrus sheep serum
LH: Luteinizing hormone
SOF: Synthetic oviduct fluid
P4: Progesterone
E2: Estradiol
RIA: Radioimmunoassay
IGF-1: Insulin-like growth factor-1
ELISA: Enzyme-linked immune sorbent assay
FBS: Fetal bovine serum
DCFH-DA: Dichlorofluorescein diacetate
ROS: Reactive oxygen species
GSH: Glutathione
MMP: Mitochondrial membrane potential
PFA: Paraformaldehyde
SDS-PAGE: SDS polyacrylamide gel electrophoresis
DEGs: Differentially expressed genes
DEPs: Differentially expressed proteins
FDR: False discovery rate
DMs: Differential metabolites
OS: Oxidant stress
NAS: N-Acetyl-5-Hydroxytryptamine
DCA: Deoxycholic acid
CDCA: Chenodeoxycholic Acid
SOD: Superoxide dismutase.
