# Supplementary material for: Growth hormone treatment improves the development of follicles and oocytes in prepubertal lambs
Source: J Ovarian Res. 2023 Jul 5;16:132. doi: 10.1186/s13048-023-01209-y (PMC10324218; doi:10.1186/s13048-023-01209-y)
Supplement: Supplementary file 4 — Additional file 4. Fig. S1. Optimize the time for GH treatment of GCs; Table S1. The information of the primary antibodies; Table S2. Primers for quantitative real-time PCR. [file 13048_2023_1209_MOESM4_ESM.docx]

**Supplementary figure**

Fig. S1 Optimize the time for GH treatment of GCs


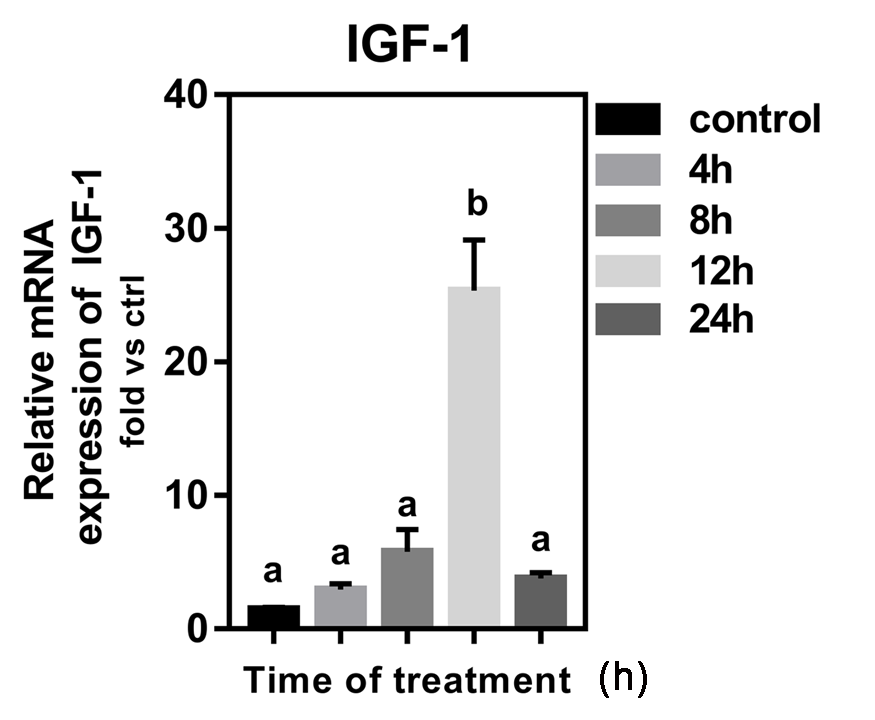


The lambs GCs were cultured in medium supplemented with 100 μg/ml GH for different hours (4 to 24 h), and then the cells were harvested for detection of the IGF1 expression by RT-qPCR.

**Table S1 The information of the primary antibodies**

| The antibody name | Catalog No. | Source | Dilution | Company |
| --- | --- | --- | --- | --- |
| β-Actin | AA128 | Mouse | WB 1:1000 | Beyotime |
| p-STAT5A | D155020 | Rabbit | WB 1:500 | Sangon |
| STAT5A | D220085 | Rabbit | WB 1:500 | Sangon |
| p-AKT | D155022 | Rabbit | WB 1:500 | Sangon |
| pan-AKT | D260001 | Rabbit | WB 1:500 | Sangon |
| BAX | AF0054 | Mouse | ICC 1:50 | Beyotime |
| BCL-2 | AB112 | Rabbit | ICC 1:50 | Beyotime |
| IGF-1 | D260510 | Rabbit | IHC 1:50 | Sangon |

**Table S2 Primers for quantitative real-time PCR**

| Genes name | Sequences (5'-3') |
| --- | --- |
| *DDIT4L* | Forward: TAGTCGAGGTCGCTTCTCCT |
|  | Reverse: CTACAATGACTTTGGGCGGC |
| *PDK4* | Forward: TGGTGTTCCCCTGAGAGTCA |
|  | Reverse: GTAACCAAAACCAGCCAGCG |
| *HTR1E* | Forward: GCTCCGTCCACAAGGTGAAAGA |
|  | Reverse: CTCAGCTGTGTCGTCTCTTGG |
| *CSMD3* | Forward: ACGTCATGTGACAACCCAGG |
|  | Reverse: TACAGCTGTGGGGTATGCAC |
| *CNTN3* | Forward: ATAGCCAAAGTGGAGCCGTC |
|  | Reverse: TTCACCCATGACACCGTCAG |
| *COL5A2* | Forward: ACGTGGGCAAGACCATCTTT |
|  | Reverse: AACTGGCCCAATTTCAACGC |
| *CSF2RB* | Forward: GCAGGTTGCTGGTTGAAGTG |
|  | Reverse: CATGCCCTTGGGTCGTTTTC |
| *OSMR* | Forward: AGAAACCGGTGTGATTGGCA |
|  | Reverse: GGAGCCAAGGGATGAACTCC |
| *LPAR3* | Forward: CCAACGTCTTGTCTCCGCAT |
|  | Reverse: CACACAACGAAGGCCCCTAA |
| *COX1* | Forward: TGGAGGACAATATCAAGGGAGGAG |
|  | Reverse: GGACCGAAACCTGAACACAACC |
| *PLA2G1B* | Forward: TGGATGATCTGGACAGGTGCT |
|  | Reverse: ACGGTCACAGTTGCAGATGA |
| *LRRC43* | Forward: CACCTACTTCAGGTCGCTCC |
|  | Reverse: ACAGTTCCAGGACCTTGAGAG |
| *CD14* | Forward: ATTGACGCTTGAGGACCTGG |
|  | Reverse: GTGCTTGGGCAATGTTCAGG |
| *HDAC1* | Forward: ACCATTTGAGAAGGGTGGCT |
|  | Reverse: TGCAGAATTGGGGGAAGACA |
| *PLPP1* | Forward: TTATAAGTAGAGGCTGGCGGC |
|  | Reverse: AAAAACCCCAGTGGATGGGC |
| *IGF-1* | Forward: TGAAGCAGGTGAAGATGCCA |
|  | Reverse: TACCCCGTGGGCTTGTTGAA |
| *CDKN1B* | Forward: GACCTGCCGCAGATGATTCC |
|  | Reverse: CTTCTGAGGCCAGGCTTCTT |
| *CDK2* | Forward: GACTTCGGACTAGCCAGAGC |
|  | Reverse: CGGGTCACCATCTCAGCAAA |
| *CDK4* | Forward: CAGTGTACAAGGCCCGTGAT |
|  | Reverse: GACGTCCATGAGCCTGACAA |
| *C-MYC* | Forward: AACTTACAACACCCGAGCGA |
|  | Reverse: CATCGCGGTAGCCCGTATTT |
| *CASPASE3* | Forward: CCGAAAGGTAGCGACAG |
|  | Reverse: GTTTGGTCACTTGGCATACA |
| *BAX* | Forward: TCTACTTTGCCAGCAAACTGGTGC |
|  | Reverse: AAGGAAGTCCAATGTCCAGCCCAT |
| *BCL-2* | Forward: TTCGCCGAGATGTCCAGTCA |
|  | Reverse: TCCGAACTCAAAGAAGGCCACGAT |
| *AKT1* | Forward: GAGACGATGGACTTCCGGTC |
|  | Reverse: CTCATTCATGGTCACGCGGT |
| *AKT2* | Forward: CCTTTCCTTGCTTCCCGAGT |
|  | Reverse: GCTGAAGTAACCTCCACCCC |
| *AKT3* | Forward: GCAGCAGCAGAGAATCCAAAC |
|  | Reverse: CTTCCCTTTACCAGCACCCCT |
| *PIK3R3* | Forward: GGCTCATTGTCTTCCTGGCT |
|  | Reverse: CTGAGCTATGGTCGAGGCTG |
| *PIK3CD* | Forward: GAACAAACACGAGGACGTGG |
|  | Reverse: GGAAAAGCTCGTCGTCCGTTA |
| *GAPDH* | Forward: TGATGACATCAAGAAGGTGGTG |
|  | Reverse: TCCTTGGAGGCCATGTAGGCCAT |
